# The diagnosis of juvenile systemic lupus erythematosus with SLICC

**DOI:** 10.1186/1546-0096-12-S1-P322

**Published:** 2014-09-17

**Authors:** Ayşenur Paç Kısaarslan, Betül Sözeri, Ruhan Düşünsel, Zübeyde Gündüz, Hakan Poyrazoğlu, Sibel Yel, Kenan Yılmaz, İsmail Dursun, Funda Baştuğ, Sebahat Tülpar

**Affiliations:** 1 Pediatric Rheumatology, Erciyes University Faculty of Medicine, Kayseri, Turkey; 2 Pediatric Nephrology, Erciyes University Faculty of Medicine, Kayseri, Turkey

## Introduction

Systemic Lupus Erythematosus (SLE) is a chronic autoimmune disease that can involve any organ system, and may lead to significant morbidity and even mortality. Childhood-onset SLE (cSLE) is a rare disease with an incidence of 0.3-0.9 per 100.000 children-years and a prevalence of 3.3-8.8 per 100.000 children.

## Objectives

SLE is called the great mimicker, as the disease shares characteristics with many other (autoimmune) diseases. Especially when the classic malar rash is absent, diagnosing SLE can be a challenge. Most patients who are diagnosed with juvenil SLE fulfill 4 or more of the American College of Rheumatology (ACR) classification criteria for SLE. The Systemic Lupus International Collaborating Clinics (SLICC) have recently suggested a new set of criteria for the classification of SLE. In recently study, SLICC criteria performed better, was more sensitive (p <0.001), and less specific (p = 0.016) than ACR criteria in childhood.

## Methods

JSLE patients (n = 83) from 2 different centers whose diagnosis fulfilled four or more of the ACR criteria were divided into two groups: those with at least one ACR mucocutaneous criterion (ACR skin feature positive) and those without (ACR skin feature negative) at diagnosis. The relative frequency of skin involvement was described by the paediatric adaptation of SLICC.

## Results

We studied 83 patients (83% female; 17% male) with SLE from two regions of Turkey. The mean age at diagnosis was 13±2,95 years. The common criteria besides ANA in ACR all patients were, respectively, haematological [n = 55 (66%)], musculoskeletal [n = 45 (54%)], and renal [n = 40 (48%)]. Fourty-five patients (54%) had ACR-defined skin involvement with no significant demographic differences compared with those without. ACR skin feature positive patients showed greater major organ involvement (haematological (68% vs 66%), renal (51% vs 45%). At the time of diagnosis, median SLICC score was 8 in ACR skin feature positive group while 6 in others. Fifty-eight per cent of ACR skin feature negative patients had skin involvement using SLICC (n=13), which included maculopapular rash (76%), toxic epidermal necrosis (0.8%), bullous rash (19%), fotosensitive rash (92%). The thirteen patients showed greater musculoscaletal, haematological and renal involvement at diagnosis (P>0.05).

## Conclusion

There are a number of other important mucocutaneous manifestations commonly found in JSLE patients apart from the four listed in the ACR criteria. These additional lesions are also associated with major organ involvement.

## Disclosure of interest

None declared

